# Machine-Learning-Enabled Design and Manipulation of a Microfluidic Concentration Gradient Generator

**DOI:** 10.3390/mi13111810

**Published:** 2022-10-24

**Authors:** Naiyin Zhang, Zhenya Liu, Junchao Wang

**Affiliations:** 1 School of Automation, Hangzhou Dianzi University, Hangzhou 310018, China; 2 Key Laboratory of RF Circuits and Systems, Ministry of Education, Hangzhou Dianzi University, Hangzhou 310018, China

**Keywords:** microfluidics, machine learning, interpolation algorithm, design automation, computer aided design

## Abstract

Microfluidics concentration gradient generators have been widely applied in chemical and biological fields. However, the current gradient generators still have some limitations. In this work, we presented a microfluidic concentration gradient generator with its corresponding manipulation process to generate an arbitrary concentration gradient. Machine-learning techniques and interpolation algorithms were implemented to help researchers instantly analyze the current concentration profile of the gradient generator with different inlet configurations. The proposed method has a 93.71% accuracy rate with a 300× acceleration effect compared to the conventional finite element analysis. In addition, our method shows the potential application of the design automation and computer-aided design of microfluidics by leveraging both artificial neural networks and computer science algorithms.

## 1. Introduction

Concentration gradient refers to the gradual change in the concentration of solutes in a solution as a function of distance through a solution. In other words, a concentration gradient is the outcome when the amount of solutes between two solutions are different. There are various concentration gradients on earth, from the giant scale, such as the dissolved oxygen concentration in ocean water from the surface to the deep zone, to the small scale, such as the famous sodium ion vs. potassium ion concentration inside and outside the cell. The word concentration usually correlates with chemical or biological molecules such as ions, atoms, and their complexes. Within the human body, there are various biomolecules being regulated by unique concentration gradients to control biological activities through cell-signaling pathways, including cell growth [1], migration [2], differentiation [3,4], immune response [5,6], wound healing [7,8], and tumor metastasis [9,10].

To understand the regulation mechanism of those concentration gradients on cell functions, scientists have developed alternative in vitro methods and tools such as different kinds of concentration gradient generators to complete the goal [11]. Previous literature reported the applications of microfluidics-based concentration gradient generators in various cell studies, such as the chemotaxis of cancer cells [11], cellular responses [11,12,13], and drug screening [14,15,16,17].

Within the above-mentioned applications, drug screening has been extensively studied in recent years due to the unique benefits of small size, cost efficiency, and the high-throughput of microfluidics. Hong et al. constructed a Christmas-tree structured microfluidic device on a paper platform to generate concentration gradients of doxorubicin (DOX) to evaluate HeLa cell viability under different DOX concentrations for high-throughput drug screening [14]. Lim et al. developed a microfluidic spheroid culture device that can generate concentration gradients of cancer drug irinotecan for the high-throughput probing of the dynamic signaling of colon cancer cell HCT116 [15]. Mulholland et al. applied repeatable concentration gradients to extensive drug screening in the presence of a limited number of available primary human prostate cancer cells, delivering a range of drug concentration simultaneously to multiple-sized spheroids [17]. Luo et al. developed a circular concentration gradient generator for high-throughput screening of drugs against type 2 diabetes, using glipizide as a model drug and the insulinoma cell line as a model cell [16].

Compared to the research on cell spheroids, Guo et al. designed a multichannel synchronous hydrodynamic gating with microfluidic concentration gradient generators to probe the dynamic signaling of single Hela cells [18]. In order to rapidly generate continuous concentration gradients of multi reagents on a millimeter-sized sample such as tissue instead of just micron-sized sample such as cells, Rismanian et al. modified the tree-like concentration gradient generator with a micromixer, and they successfully achieved the simultaneous delivery of two reagents on a millimeter-sized sample [19]. Compared to a single-layered chip, Yang et al. constructed a microfluidic concentration gradient generator with parallel multi-channels and multi-layers to produce multi-color nanoparticles, which could be adapted for synthesizing desired metallic nanoparticles for research applications [20]. Tang et al. developed multi-layered centrifugal microfluidics that could generate linear concentration gradients to test the antimicrobial susceptibility of ampicillin against *E. coli* [21]. Yang et al. developed a microfluidic concentration gradient chip with a radial channel network for HeLa cell apoptosis analysis, and the concentration ranges generated over hundreds of branches were wide and predictable [12]. Sugiyama et al. studied the proactive role of a cell membrane in regulating molecular transport against the concentration gradient [13].

The current studies have presented new solutions for many research areas. However, there are still challenges preventing microfluidic gradient generators from a easier and more applicable use. Firstly, the biomolecule concentrations inside the human body are in a great range while most of the current microfluidic concentration gradient generators can only generate a narrow and fixed-range of concentration gradient. Secondly, the concentration gradient generated in the device is commonly determined by the inflow concentration, which is difficult for researchers to manipulate or program. Thirdly, the waste liquid generated from the device remains a significant part. Last but not least, the fluid flow from outlets is usually assumed in an ideal state, without considering the effects of downstream structures, which might be crucial for biological applications. During the design process, the velocity field and concentration profile are the key parts and can be simulated using finite element analysis (FEA) [22]. However, the simulation process is time-consuming, and the matching consistency between limited pre-generated designs and user desire is not stable [23]. To address the above issues, machine-learning techniques provide a promising solution [24,25]. In our previous work, we used convolutional neural networks to predict both the concentration and velocity behavior of all three outlets for random microfluidic mixers [26].

In this work, we take a further step and propose a microfluidic device, which can generate an arbitrary concentration gradient based on different inlet configurations in a fixed 2 mm × 8 mm region for biological and chemical applications. With the help of supervised learning [27], the boundary condition of the concentration profile was predicted by an artificial neural network, and an interpolation algorithm was implemented afterwards to generate the complete concentration profile for the whole reactive region instead of purely outlets. As a result, only a one-second process time is needed for a normal desktop (Intel i5 CPU with 8 GB RAM) to predict one typical target design, with an acceptable accuracy rate (around 90% to 95%) compared with conventional FEA.

## 2. Theory of the Design

**Figure 1:**
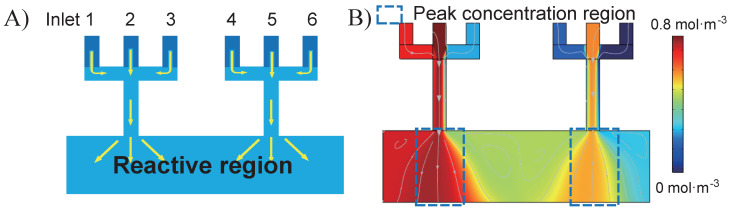
(**A**) The schematic of the proposed gradient generator, which has six inlets and one reactive region. (**B**) One demonstrated example with randomly generated inlet configurations, which has two peak concentration regions and three valley concentration regions.

The schematic of the proposed gradient generator is shown in Figure 1A, which has six inlets and one 2 mm × 8 mm reactive region, with inlets width all set to 0.4 mm. The linear flow rate of all six inlets varies from 0 mm$\cdot s^{-1}$ to 1 mm$\cdot s^{-1}$, while the corresponding concentration varies from 0 mol$\cdot m^{-3}$ to 1 mol$\cdot m^{-3}$. By manipulating the values of all twelve parameters (six flow rates and six concentrations) of the inlets, different concentration profiles in the reactive region can be achieved. For instance, Figure 1B is one demonstrated example with randomly generated inlet configurations, which are listed in Table 1.

Unlike most other gradient generators, whose highest concentration starts from one end and whose lowest concentration ends up at the other end, the proposed gradient generator has two peak concentration regions and three valley concentration regions. This set-up provides the reactive region more opportunities to achieve assorted concentration profiles for different applications. Specifically, the concentration profile of the proposed example shows two peak fluxes in the reactive region, which are 0.8 mol$\cdot m^{-3}$ and 0.6 mol$\cdot m^{-3}$. In contrast, there are three valley fluxes around the two peak fluxes, whose values are 0.7 mol$\cdot m^{-3}$, 0.5 mol$\cdot m^{-3}$, and 0.3 mol$\cdot m^{-3}$.

## 3. Materials and Methods

### 3.1. Building a Database of the Concentration Gradient Generators with Different Inlet Configurations

In this work, supervised learning [27] is used to train an artificial neural network (ANN) to replace the FEA for predicting the upper and bottom boundaries of the reactive region. Thus, the very first step is to build a database of the concentration gradient generators with various inlet configurations, which will be used as the training dataset in the following steps.

**Figure 2:**
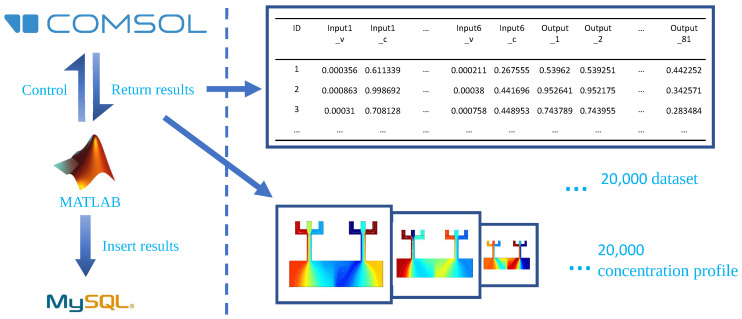
The overall flow of automating COMSOL to prepare the dataset of the proposed gradient generator with different inlet configurations. The linear flow rate of all six inlets varies from 0 mm$\cdot s^{-1}$ to 1 mm$\cdot s^{-1}$, and the inflow concentration varies from 0 mol$\cdot m^{-3}$ to 1 mol$\cdot m^{-3}$.

As shown in Figure 2, the behaviors of the proposed gradient generator were simulated using the FEA software COMSOL Multiphysics 5.5 (COMSOL Inc., Burlington, MA, USA). The whole process was automated with MATLAB 2020a by using an official API (LiveLink for MATLAB) provided by COMSOL. The velocity field of each gradient generator configuration was simulated in the laminar flow (LF) physics module in COMSOL multiphysics, and the concentration profile was simulated in the Transport of Diluted Species (TDS) physics module. A stationary solver was used to compute both LF and TDS physics. Besides, mesh independence was investigated. The value of the diffusion coefficient was set to $4.25\times 10^{-10}\;m^{2}$·$s^{-1}$. More detailed simulation parameters can be found in Table 2.

After varying the value of all twelve parameters of inlets in a certain range (as mentioned in the above section), totally 20,000 different inlet configurations of the proposed gradient generator were investigated. The corresponding dataset and concentration profiles were stored in a local MySQL database. The dataset was used as the training and test set in the following ANN training process. And the COMSOL-predicted concentration profiles were used as the standard to be compared with the concentration profiles generated by our proposed method.

### 3.2. Training an ANN for Predicting the Two Boundaries of the Reactive Region

**Figure 3:**
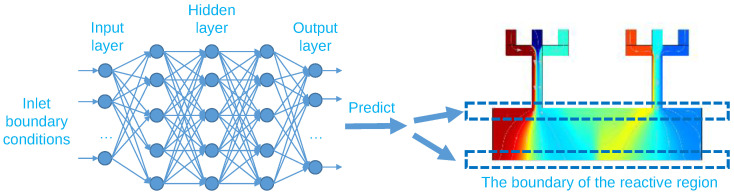
The flowchart of the proposed ANN.

As shown in Figure 3, to train the target ANN, twelve parameters of inlet boundary conditions were used as input, while the concentration of upper and bottom boundaries of the reactive region were used as output. To capture the behavior of the two boundaries, 81 points on each boundary were taken as samples with a 0.1 mm gap between each other. Therefore, totally 162 (81 + 81) data points were applied to the output layer. The details of the configuration of the proposed ANN were clarified in Table 3.

The training process was implemented in Python 3.10 with the help of PyTorch. Mean squared error (MSE) was used to calculate the loss during the training process, whose formula is described in Euqation (1).

(1) $$Loss=\frac{1}{n}\sum_{i=1}^{n}\frac{1}{162}\sum_{j=1}^{162}{\left(C_{i,j}-C_{i,j}^{\prime }\right)}^{2}$$

where *n* indicates the total items in the training set, which is 70% × 20,000 = 14,000; 162 indicates 81 sampling points on the upper boundary and 81 sampling points on the bottom boundary; $C_{i,j}$ indicates the concentration values predicted by COMSOL; and $C_{i,j}^{\prime }$ indicates the concentration values predicted by the ANN in the training process. The calculation process was implemented in Python by using the built-in PyTorch loss calculation function (*torch.nn.MSELoss*). In addition, the accuracy rate was determined by using R Square [28], whose values were determined by the built-in Python accuracy calculation function (*torchmetrics.R2Score*).

### 3.3. Completion of the Concentration Profile by Interpolation Algorithm

**Figure 4:**
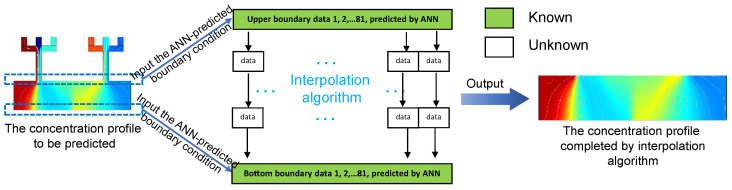
The flowchart of completing the unknown data points of the reactive region based on the linear interpolation algorithm.

Interpolation is a type of estimation, a method of constructing new data points based on the range of a discrete set of known data points [29]. As shown in Figure 4, the data points on the upper and bottom boundaries were used as known data points to predict all the unknowns between them, and the unknowns were defined into a Numpy array. Then, a two-dimensional linear interpolation algorithm was implemented by using the official Numpy package in Python 3.10 [30] to complete the concentration profile of the reactive region, which was finally ready for researchers to analyze.

## 4. Results and Discussion

### 4.1. Training of the Proposed ANN

**Figure 5:**
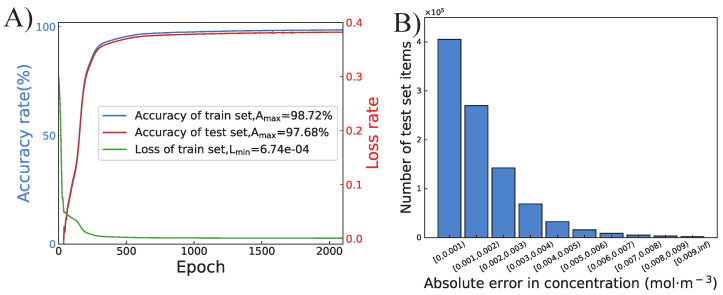
(**A**) The training curve of the proposed ANN during 2000 epochs. (**B**) The histogram of the absolute error in concentration of each data point on two boundaries from test set.

The training process and the performance of the proposed ANN are shown in Figure 5. Seventy percent of 20,000 datasets are used as the training set, and the other thirty percent are used as the test set. As we can see from Figure 5A, the loss rate is 6.74 $\times \;10^{-4}$, the accuracy rate of the training set is 98.72%, and the accuracy rate of the test set is 97.68% after training of 2000 epochs. Figure 5B shows the absolute error in concentration from the test set. The absolute errors of over 40%, 27%, and 13% of the concentration data points on two boundaries are less than 0.001 mol$\cdot m^{-3}$, 0.002 mol$\cdot m^{-3}$, and 0.003 mol$\cdot m^{-3}$, respectively. The error of less than 0.5% of the predicted data points is larger than 0.009 mol$\cdot m^{-3}$, which is still a relatively small error for actual experiments. This indicates high consistency between the COMSOL-predicted concentration and ANN-predicted concentration.

**Figure 6:**
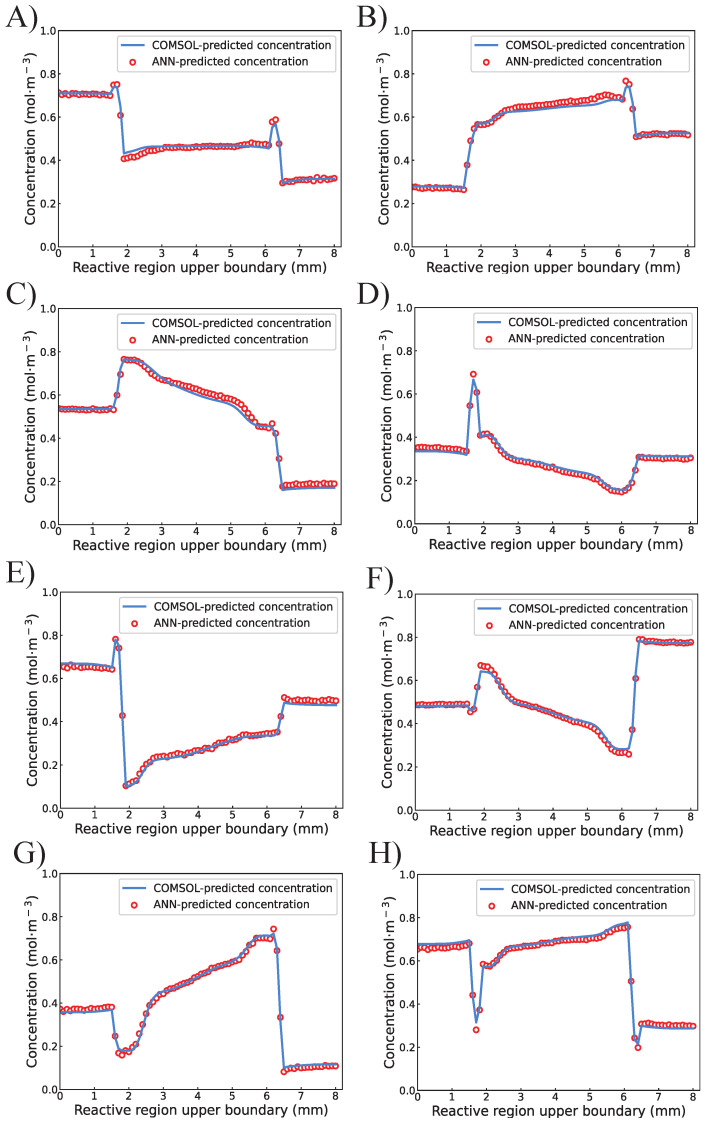
The comparision between ANN-predicted and COMSOL-predicted concentration values on the upper boundary of the reactive region from design (**A**–**H**).

**Figure 7:**
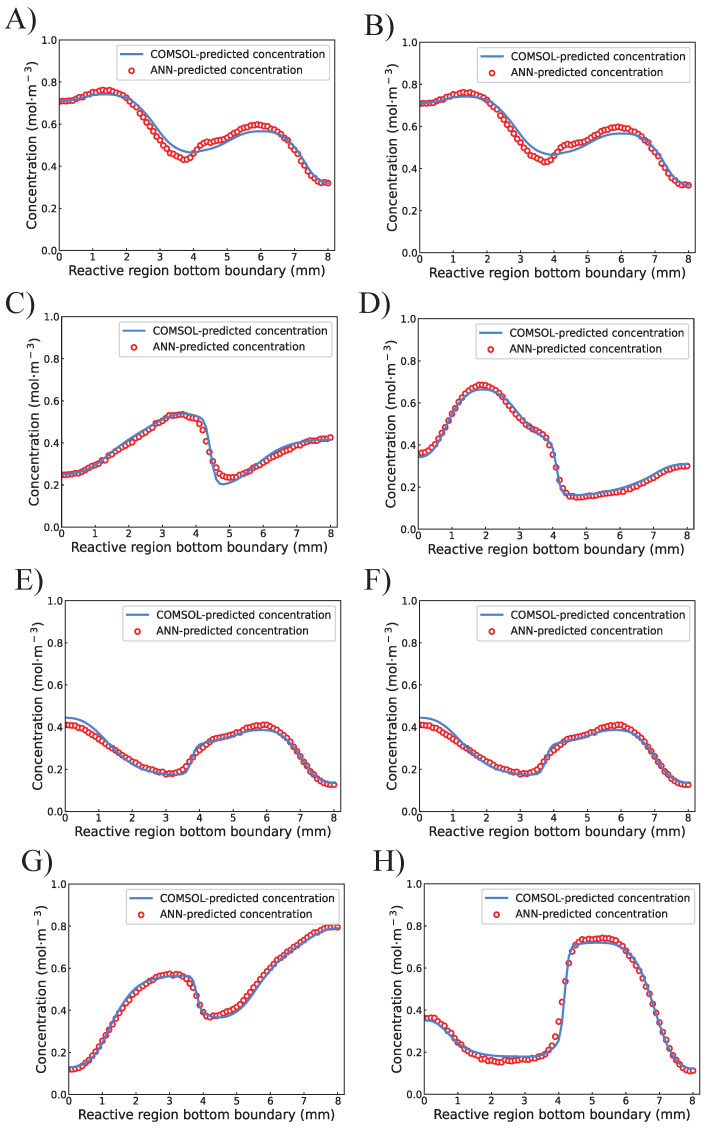
The comparision between ANN-predicted and COMSOL-predicted concentration values on the bottom boundary of the reactive region from design (**A**–**H**).

To further investigate the actual performance of the proposed ANN, eight designs (A to H) were randomly selected from the test set. The comparison between ANN-predicted concentration and COMSOL-predicted concentration values of 81 points on the upper boundary is visualized in Figure 6. It is clear that most data points predicted by the proposed ANN are right-matched with the results predicted by COMSOL. Only a small number of the ANN-predicted points are not covering the COMSOL-predicted results but are still standing closely. Similar results and conclusions can be drawn from Figure 7, which indicates high consistency between the COMSOL and ANN for predicting the concentration values on the bottom boundary.

### 4.2. Completion of the Concentration Profile by Interpolation Algorithm

**Figure 8:**
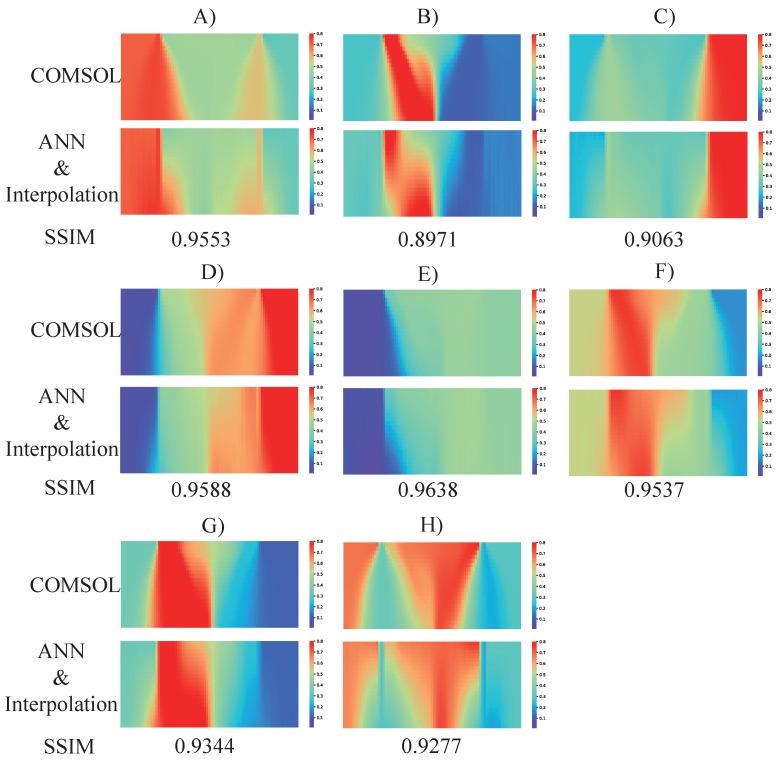
The concentration profile of the reactive region predicted by COMSOL and proposed ANN and interpolation method. The corresponding SSIM values are listed for quantification analysis as well between two methods. The SSIM values of design (**A**–**H**) are 0.9553, 0.8971, 0.9063, 0.9588, 0.9638, 0.9537, 0.9344, and 0.9277, respectively.

As mentioned in the above section, the proposed ANN is able to predict the upper and bottom boundary of the concentration of the reactive region with 97.68% accuracy. Then, a two-dimensional linear interpolation algorithm was implemented to achieve a complete map of the concentration profile of the reactive region. To investigate the performance from the combination of ANN prediction and interpolation algorithm, the results from the same eight designs are shown in Figure 8. It is quite interesting that the concentration profiles from all eight designs between COMSOL and ANN predictions show high-level similarities. In addition, the predicted (ANN + Interpolation) concentration profiles from design C, D, E, F, and G look almost identical to COMSOL ones from the view of human beings.

To quantify the similarity from a statistical point of view, image similarity analysis was performed on all eight designs. The algorithm we used is the structural similarity index measure (SSIM), which has been widely applied for measuring the similarity between two images in many different fields [31,32]. The SSIM values of all eight designs (A to H) are 0.9553, 0.8971, 0.9063, 0.9588, 0.9638, 0.9537, 0.9344, and 0.9277, respectively, with an average value of 93.71%. Except for the fact that the SSIM value of design B is less than 0.9, the values of all the other cases are greater than 0.9, which indicates a successful concentration completion process of our proposed method.

In microfluidic situations, the Reynolds number is usually smaller than 1, which indicates a laminar flow physics with fluid particles following smooth paths in different layers. This makes the proposed linear interpolation algorithm suitable for our scenes. However, if the Reynolds number increases and the laminar flow becomes turbulent flow, the interpolation algorithm shall fail. In other words, the concentration profile can be predicted based on the boundary concentration in low Reynolds number conditions instead of high Reynolds number conditions.

Though our proposed method cannot achieve a 100% accuracy rate with finite element analysis provided by COMSOL, the overall computational time is reduced from around 5 to 10 min to less than one second, which has at least 300× acceleration effect in a normal desktop.

## 5. Conclusions

This work has presented a microfluidic concentration gradient generator with its corresponding manipulation process to generate an arbitrary concentration gradient. With the help of machine-learning techniques and interpolation algorithms, a brand new concentration profile can be provided in less than one second to facilitate users to analyze and determine whether to apply the new settings. The trade-off is the accuracy rate of our proposed method is 93.71% on average (based on SSIM), which is still acceptable considering the over 300× acceleration effect. In addition, to present a new type of microfluidic concentration gradient generator, our proposed method provides an example and shows the inherent potential of the design automation of microfluidics by leveraging both ANN and computer algorithms.

### Potential Limitations

Since our proposed methods are based on the combination of an ANN and interpolation algorithm, two major limitations are mainly come from the limitations of both algorithms. Firstly, if the geometry of the gradient generator is changed or modified, the pre-trained ANN shall be re-trained by the newly generated dataset. This limitation can be partially addressed by applying the transfer learning algorithm with the recycle use of the pre-trained ANN [33]. Secondly, thanks to the low Reynolds number (Re ≪ 1) situation in microfluidics, the linear interpolation algorithm works reasonably well. However, as the Reynolds number increases (Re ≫ 1), a turbulent phenomenon might intervene and make the interpolation algorithm invalid [34,35].

## Figures and Tables

**Table 1:** The configuration of inlet boundary conditions for the demonstrated example in Figure 1B.

N/A	Inlet 1	Inlet 2	Inlet 3	Inlet 4	Inlet 5	Inlet 6
Flow rate (mm$\cdot s^{-1}$)	0.68	0.87	0.41	0.15	0.91	0.28
Concentration (mol$\cdot m^{-3}$)	0.69	0.77	0.29	0.22	0.59	0.083

**Table 2:** Simulation parameters in COMSOL.

Parameter	Value
Inlet flow rate	Randomly generated from 0 to 1 mm$\cdot s^{-1}$
Inflow concentration from six inlets	Randomly generated from 0 to 1 mol$\cdot m^{-3}$
Diffusion coefficient	$4.25\times 10^{-10}\;m^{2}$·$s^{-1}$
Mesh max element size	175 μm
Mesh min element size	5 μm
Mesh max element growth rate	1.13
Mesh curvature factor	0.3
Mesh resolution of narrow regions	1
Tolerance to convergence	0.001

**Table 3:** The configuration of the proposed artificial neural network.

Layer	Type	Depth	Activation
1	Input layer	12	Linear
2	Fully-connected layer	120	ReLU
3	Fully-connected layer	120	ReLU
4	Fully-connected layer	120	ReLU
5	Output layer	162 (81 + 81)	Linear

## Data Availability

Not applicable.
